# Continuous palliative sedation for patients with advanced cancer at a tertiary care cancer center

**DOI:** 10.1186/s12904-017-0264-2

**Published:** 2018-01-04

**Authors:** Bernard Lobato Prado, Diogo Bugano Diniz Gomes, Pedro Luiz Serrano Usón Júnior, Patricia Taranto, Monique Sedlmaier França, Daniel Eiger, Rodrigo Coutinho Mariano, David Hui, Auro Del Giglio

**Affiliations:** 1 Oncology Department, Hospital Israelita Albert Einstein, 627 Albert Einstein Av., Sao Paulo, 05652-900 Brazil; 2 Department of Palliative Care and Rehabilitation Medicine, The University of Texas MD Anderson Cancer Center, 1515 Holcombe Blvd, Houston, USA; 3 Faculdade de Medicina do ABC, 821 Principe de Gales Av, Santo André, Brazil

**Keywords:** Continuous palliative sedation, Advanced cancer, Tertiary care, Midazolam, Neuroleptics, Survival

## Abstract

**Background:**

Palliative sedation (PS) is an intervention to treat refractory symptoms and to relieve suffering at the end of life. Its prevalence and practice patterns vary widely worldwide. The aim of our study was to evaluate the frequency, clinical indications and outcomes of PS in advanced cancer patients admitted to our tertiary comprehensive cancer center.

**Methods:**

We retrospectively studied the use of PS in advanced cancer patients who died between March 1st, 2012 and December 31st, 2014. PS was defined as the use of continuous infusion of midazolam or neuroleptics for refractory symptoms in the end of life. This study was approved by the Research Ethics Committee of our institution (project number 2481–15).

**Results:**

During the study period, 552 cancer patients died at the institution and 374 met the inclusion criteria for this study. Main reason for exclusion was death in the Intensive Care Unit. Among all included patients, 54.2% (*n* = 203) received PS. Patients who received PS as compared to those not sedated were younger (67.8 vs. 76.4 years-old, *p* < 0.001) and more likely to have a diagnosis of lung cancer (23% vs. 14%, *p* = 0.028). The most common indications for sedation were dyspnea (55%) and delirium (19.7%) and the most common drugs used were midazolam (52.7%) or midazolam and a neuroleptic (39.4%). Median initial midazolam infusion rate was 0.75 mg/h (interquartile range – IQR - 0.6-1.5) and final rate was 1.5 mg/h (IQR 0.9–3.0). Patient survival (length of hospital stay from admission to death) of those who had PS was more than the double of those who did not (33.6 days vs 16 days, *p* < 0.001). The palliative care team was involved in the care of 12% (*n* = 25) of sedated patients.

**Conclusions:**

PS is a relatively common practice in the end-of-life of cancer patients at our hospital and it is not associated with shortening of hospital stay. Involvement of a dedicated palliative care team is strongly recommended if this procedure is being considered. Further research is needed to identify factors that may affect the frequency and outcomes associated with PS.

**Electronic supplementary material:**

The online version of this article (10.1186/s12904-017-0264-2) contains supplementary material, which is available to authorized users.

## Background

Advanced cancer patients have a high symptom burden during the trajectory of their diseases, especially in the end-of-life when many of them suffer from pain, dyspnea, and fatigue [1–3].

Palliative sedation (PS) is an ethical and well accepted medical intervention defined as the deliberately use of sedative medications to reduce patients’ consciousness in order to manage end-of-life symptoms that have become refractory and intolerable [4, 5]. It is a clearly distinct practice from that of euthanasia and physician-assisted suicide, both characterized by having different concepts, intentions, procedures, and outcomes [6, 7].

The prevalence of PS varies considerably worldwide (1–88%), mainly because of differences in PS definitions, care settings and in clinical practice patterns. Mixed cases in similar settings, the degree of adherence to PS guidelines and the level of expertise of health care professionals may also play a role for this wide range [8–10].

Distinct classes of drugs are recognized as appropriate for PS; however the benzodiazepines, in particular midazolam, are the most frequently used for this purpose. PS sedatives can be administered intermittently or continuously, through a subcutaneous or an intravenous route and in a variety of clinical settings, determining different levels of sedation (mild or deep) to achieve adequate symptom relief [11–13].

Delirium and dyspnea, common symptoms in the advanced cancer population, are the leading causes of PS, although there is substantial interstudy variability on this issue. If indicated appropriately and applied judiciously, the best available evidence suggests that PS does not hasten death [14–16].

In our institution, a PS Policy was established in January 2009 and revised on March 2012, providing guidance on the clinical use of PS [17]. The aim of our study was to evaluate the frequency, clinical indications and outcomes of PS in advanced cancer patients admitted and deceased at our tertiary comprehensive cancer center.

## Methods

This study was approved by the Research Ethics Committee of our institution (project number 2481–15), a 657-bed tertiary general hospital. During 2014 we have had 52.103 discharges and about 4% of them were from oncology/hematology patients.

Palliative care activities started in 2011 at our institution, predominantly offering care to the oncology/hematology inpatient population by a mobile consultation service. Currently, three physicians and a nurse provide palliative and supportive care for all the inpatient population. The palliative care team works in straight relation with the primary team in order to manage physical and psychological symptoms, providing illness understanding to patients and its relatives and helping in care coordination and planning. Psychologists, social workers, nutritionists, pharmacists, physical and occupational therapists are also available for consultation if needed.

### PS policy and definition

Our PS institutional policy describes the definition of PS, its main clinical indications as well as recommends drugs and doses to be used. PS can be practiced in wards, intensive care units (ICU) or in the step-down units (SDU) of our hospital. Although advised in our policy, the involvement of a palliative care specialist is not mandatory. Our policy is provided as Additional file 1 [17].

PS definition was based on our 2012 institutional reviewed policy and on the National Academy of Palliative Care Handbook (NAPCH) published in August 2009 [17, 18]. These two documents define PS as the use of sedative drugs to reduce patient’s consciousness with the intent of relieving refractory symptoms during the last hours or days of a progressive and incurable disease. After refractoriness is determined, dyspnea, psychomotor agitation and terminal delirium, pain, nausea and massive bleeding are listed reasons for which PS is recognized as appropriate. Our policy points out the need of obtaining consent from the patient, if he/she is capable of making decisions for themselves, or from his/her legal representative in the case of medical incapacity. It also states that proceeding with PS should be preceded by a thorough discussion with the multidisciplinary team, the patient, family members, and caregivers. The decision about the suspension of medications and/or interventions relies on a discussion between providers and the patient/family unit.

NAPCH and our PS policy recommend midazolam and neuroleptics such as chlorpromazine as the drugs of choice to be used continuously if a more deep sedation is needed. Based on this we studied the use of continuous infusion (subcutaneous or intravenous) of midazolam and/or neuroleptics for refractory symptoms in the end-of-life of advanced cancer patients that died at our hospital.

### Patients and procedures

Five hundred fifty-two consecutive cancer patients that were admitted to our hospital and died during hospital stay between March 1st, 2012 and December 31st, 2014 were identified. Their medical records were then retrospectively reviewed to collect data of those who received PS as defined by our guidelines. Non-advanced cancer patients, those that were sedated for other purposes (e.g.: surgical procedures, endoscopy, mechanical ventilation, anesthesia, etc.), or for whom there was no clear registration of PS indications and procedures in their charts were excluded. Patients who received intermittent PS or the administration of oral sedatives were excluded. Also, we excluded ICU patients because they frequently receive sedation for other purposes like mechanical ventilation, invasive procedures, surgeries and invasive exams. We considered that in a chart review study of retrospective nature, this would be a main confounding factor that would be difficult to assess clearly.

We obtained data on PS features and details, including its indications, duration, and infusion rates of studied sedatives. As opioids are commonly maintained during PS to control dyspnea and pain we searched for information on opioid usage to identify its type and to calculate the morphine equivalent daily dose of each one of them in the last 24 h of life. Corticosteroids and neuroleptics administration at the time of PS or 24 h before its start, and the maintenance of artificial nutrition or hydration during PS were also evaluated. PS duration was defined as the time from initiation of midazolam or chlorpromazine to death. Patient survival was measured as the length of hospital stay from admission to death.

Patient’s general demographic characteristics, cancer histology/tumor type, reason for admission and place of death (ward or step down unit) were also analyzed.

Finally, and considering that PS can be practiced by different specialties in our institution, we decided to identify which primary team was leading care and which physician specialty initiated the intervention (oncology, hematology, surgery, palliative care or other medical specialties).

### Statistical analyses

Patient demographics were reported for sedated and non-sedated patients and for each subgroup of sedation. For categorical variables, we performed a Chi-squared test; for continuous data, we used ANOVA or the Kruskal-Wallis test, depending on data distribution.

Survival analysis was measured from date of admission to date of death or from date and hour of sedation to death. Patients were censored at last follow-up. We plotted traditional Kaplan-Meier survival functions and used a Cox regression model to compare groups. The rate of missing data was lower than 5% for the studied variables and, therefore, no adjustment for missing data was needed.

We used Stata SE version 10.3 (StataCorp, College Station, Texas) for all analysis and significance was set at 0.05, unless otherwise stated.

## Results

**Fig. 1:**
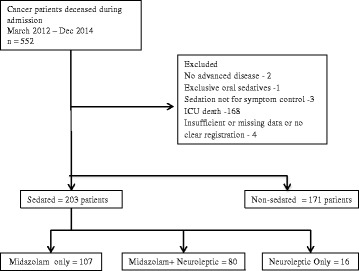
Diagram of the study population

Between March 1st 2012 and December 31st 2014, a total of 374 patients met the inclusion criteria for this study. The main reason for exclusion was ICU death (*n* = 168). Among all included patients, 54.2% (*n* = 203) received PS. Midazolam or midazolam combined with a neuroleptic were the most frequent prescribed sedatives for PS (Fig. 1).

### Demographic and survival differences between sedated and non-sedated patients

**Fig. 2:**
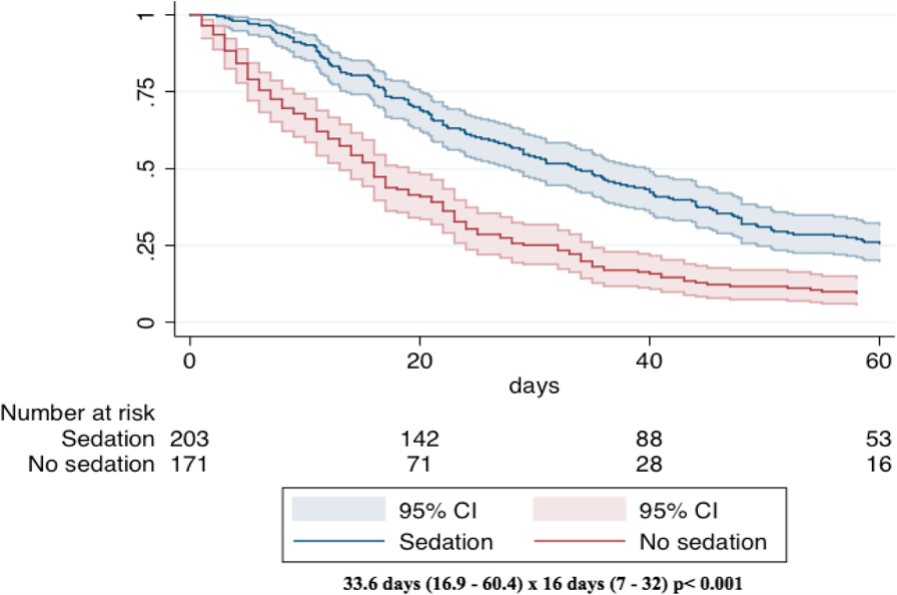
Time from hospital admission to death

Table 1 describes the differences between sedated and non-sedated patients. Those who received PS were younger (67.8 vs 73.34 years-old, *p* < 0.001), more likely to have a primary diagnosis of lung cancer (23% vs 14% *p* = 0.028) and to be seen by oncology/hematology rather than other medical or surgical specialties (71% vs 45.1% *p* < 0.001) compared with patients that did not receive sedation. Patient survival, measured as the length of hospital stay from admission to death of those who had PS, was more than the double of those who did not (33.6 days vs 16 days p < 0.001) as shown in Fig. 2.

**Table 1:** Demographic and clinical differences between sedated and non-sedated patients

Characteristics	Sedated	Non-sedated	*p* value
Number	203	171	
Age – years (mean, SD)	67.8 (15.2)	76.4 (15.4)	<0.001
Male (%)	44.3	49.1	0.321
Place of death			
Regular wards	168 (82%)	137 (80%)	0.477
Step-down unit	35 (17%)	34 (20%)	
Primary team – n (%)			
Oncology/Hematology	145 (71%)	78 (45.6%)	<0.001
Other medical	41 (20.6%)	78 (45.6%)	
Surgery	15 (7.4%)	14 (8.1%)	
Others	2 (1%)	1 (0.6%)	
Cancer type- n (%)			
Lung	47 (23%)	25 (14%)	0.028
Hepatobiliary	13 (6%)	19 (11%)	
Breast	16 (8%)	14 (8%)	
Gastroesophageal	10 (5%)	8 (5%)	
Colorectal	11 (5%)	10 (5%)	
Pancreas	11 (5%)	7 (4%)	
Lymphoma/leukemia	8 (4%)	15 (10%)	
Gliomas	11 (5%)	8 (5%)	
Others	76 (39%)	65 (40%)

### Characteristics of the sedated population

General demographic and clinical characteristics of the sedated population are shown in Tables 1 and 2. The mean age was 67.8 years and 55.4% were women. As expected, the vast majority had metastatic disease (88.7%) and the main reason for admission was an infection (23%). Lung cancer was the most common cancer diagnosis (23%). Oncology/hematology specialties led patient care in 71% of the cases. A dedicated Palliative Care Team was involved in patient care in only 25 patients (12.3%). The most common indications for sedation were dyspnea (55.1%) and delirium (19.7%).

**Table 2:** Characteristics of sedated patients

Patients characteristics (n)	203
Seen by palliative care team – n (%)	25 (12.3%)
Disease stage – n (%)	
Locally advanced	3 (1.5%)
Metastatic	180 (88.7%)
Incurable^a^	20 (9.8%)
Admission – n (%)	
Infection	47 (23.5%)
Dyspnea	45 (21.5%)
Pain	22 (11%)
Bowel obstruction	13 (6.5%)
Venous thromboembolism	3 (1.5%)
Other	73 (36%)
Reason for sedation – n (%)	
Dyspnea	112 (55.1%)
Delirium	40 (19.7%)
Pain	30 (14.8%)
Bleeding	4 (2%)
Anxiety	3 (1.5%)
Other	3 (1.5%)
Not documented	11 (5.4%)

^a^Deemed incurable (e.g: local recurrence not amenable to curative treatment)

### Details of PS

Details of PS are presented in Additional file 2. Midazolam only and midazolam combined with a neuroleptic were the most frequently used sedatives for PS in 107 (52.7%) and 80 (39.4%) patients, respectively. Neuroleptics only were administered for PS in 16 patients (7.9%). Median initial midazolam dose was 0.75 mg/h (0.6–1.5) and final dose was 1.5 mg/h (0.9–3.0). Median initial chlorpromazine dose was 1.4 mg/h (1–3) and final dose was 2 mg/h (1–4). 98% of patients received at least one opioid and the most common one was morphine in 93% of them. The median dose of opioids in the last 24hs of life was 48 (24–105) mg of the morphine equivalent daily dose. Artificial hydration and nutrition were maintained during PS in 62 (20.5%) and 28 (13.8%) patients, respectively. Steroids were prescribed for about a third of the PS population (32%). There were no significant differences in practice or outcomes between patients seen by palliative care and the other groups, but there was a trend towards use of lower doses of opioids (median 40 mg; 24-64 mg vs 48mgs;24-109 mg), midazolam (1.2 mg/h 0.6;2.2 mg/h vs 1.5 mg/h; 1–3.4 mg/h) and chlorpromazine (1.2 mg/h; 1–2.0 vs 2.0 mg/h; 1–4.4) by patients seen by the palliative care team as well as a non-significant longer sedation time in this group (36.9 h; 17.6–52.5 vs 26.1 h; 12.2–58; *p* = 0.47).

Median time from admission to death was 33.6 days (17–61.7) and from sedation to death was 27 h (5.5–66.2). The group receiving neuroleptics only had the shortest time from sedation to death (8.66hs; 5.5–19.4 vs 27.9 h; 13.3–48.5hs for the midazolam group; *p* = 0.11). This group was otherwise similar to the other sedative groups regarding diagnosis, reasons for sedation and other drugs used.

## Discussion

This retrospective study provides relevant information on PS practice patterns and characteristics in an advanced cancer population of a tertiary cancer center and represents one of the largest cohorts to date. The use of PS to manage refractory symptoms in the end-of-life of cancer patients was a relatively common intervention in our setting as we found a PS frequency of 54.2%. This is somewhat comparable to that found by others. For example, Mercadante et al. found a 54.5% frequency of PS in a prospective cohort of 77 terminally ill cancer patients admitted to an acute pain relief and palliative care unit [19]. Similarly, Kohara et al. reported that half of the cancer patients admitted to a Japanese palliative care unit had their refractory symptoms managed with PS [20]. On the other hand, others had described percentages that were as high as 64% [21] or as low as 12% [22]. We believe that this great variability could be explained by considerable differences in many factors involving PS administration: patient population, clinical settings, PS definition and methods, the degree of experience with PS and adherence with current guidelines, cultural aspects and PS institutional policies [21, 23, 24]. All of these may be responsible for the wide range of PS prevalence found in the literature [8–10].

In our study, sedated patients were more likely to be younger and have a diagnosis of lung cancer. Particularly, age differences in PS prevalence and a more frequent use of the procedure in younger patients seem to be common findings in several studies. This possibly reflects the aggressive behavior of some types of tumors in these individuals, the intense treatment received by them and the complexity of clinical situations presented by a younger population [25, 26].

Many lung cancer patients admitted because of severe dyspnea developed refractory dyspnea during the hospital stay, triggering the use of PS to manage it. It is important to note that pain was not one of the leading symptoms requiring PS. In fact, only 14.8% of the sedated patients had PS because of pain. As almost all sedated patients were on opioids in the last days of life, administered doses (median MEDD = 48 mg in the last 24 h) seemed to be sufficient to maintain pain control for the majority without requiring PS.

Benzodiazepines, specifically midazolam, and neuroleptics such as chlorpromazine and haloperidol are the two most frequent types of sedatives used for PS [5, 11, 24]. We found a similar practice pattern in our study. Median initial and final midazolam doses found were also within the previously reported range and in conformity with current consensus [9, 11, 27]. In addition to sedatives, we evaluated the concomitant use of other medication (opioids and steroids) and interventions (artificial nutrition and hydration) 24 h before and during PS, detailing PS and end-of-life practicing in our setting. As expected, the vast majority of sedated patients were on opioids, a common class of drugs used for symptom management in the end-of-life, markedly for pain and dyspnea. Steroids were prescribed for one-third of the sedated population, while artificial nutrition and hydration were maintained for one-fifth or less of them. Most guidelines on PS strongly recommend continuing with previously prescribed symptom management medication during the sedation period, particularly analgesics and co-analgesic drugs. As for life-sustaining therapies they should be considered a separated decisional process to be discussed with patients, family members and caregivers [4, 5, 11].

There has always been considerable controversy surrounding PS in many aspects, but in particular regarding the concern of its possible detrimental effect on survival. Our study did not find any negative impact on patient survival among those who had PS when compared to the group of non-sedated patients. Actually, the survival time measured as the number of days from admission to death was significantly longer for the sedated population. This was a similar finding of what Mercadante et al. found in their palliative care unit cohort, although our hospital stay time was in general much higher [19]. Data found by us in this issue adds to that of the published literature. Two systematic literature reviews of retrospective and prospective studies, one involving 621 sedated patients [14], and the other involving 1137 patients who had PS [24], together with the recent published secondary analysis of the prospective J-Proval study [15], comprise the best available evidence favoring the concept that PS does not hasten death if performed adequately and following guidelines recommendations.

In our view, the long hospital stay of the sedated population found in this study could be explained by two main reasons that in fact are interconnected to each other. First, patients in this group were the most demanding and complex ones, developing challenging clinical and psychosocial situations in the end-of-life that required aggressive symptom management and intensive family/caregiver support. Because of this, the healthcare team along with family members might have decided to maintain these patients admitted to the inpatient unit, where they could be assisted rapidly and in a comprehensive manner. Second, this finding could be directly linked to the relatively limited availability of hospice services in our country, a care model that permits to offer good quality end-of-life care at home or in inpatient facilities [28–30].

The mean duration of PS in our study population was not much disparate from what has been already reported [10, 21, 25] on average 1–2 days of sedation until death, however when we did a subgroup analysis separating patients by type of sedative received we found a trend towards a statistically significant difference for a shorter sedation time in those who had only neuroleptics. The meaning of this finding and its reasons are difficult to point out because of the low number of patients in this group and further larger studies should be done to more profoundly understand the outcomes differences between distinct classes of sedatives used for PS.

In general, the recommendations of our policy were followed, specifically regarding PS indications, main drugs and doses to be used. On the other hand, even though recommended by international guidelines, the participation of a dedicated palliative care team during PS was low (12%). There are several reasons that might explain this low involvement. Firstly, the palliative care team had just started during the time frame that our study was done and many health care providers of our institution were not totally aware of the activity of the team and its potential benefits for patients and caregivers. Our policy advises that a PC specialist can be consulted, but this is not mandatory as it is in institutions from other countries [17, 23]. Thirdly and most importantly, this probably reflects the low level of palliative care development of our country, where its provision, education, public awareness and general activities are isolated, resulting in misguided concepts and maintaining developmental barriers in financial, social, political and cultural aspects [28–30]. Further efforts are needed to integrate palliative care in our country and at our institution [31].

This retrospective study has some limitations. We did not have a regular and standardized method for symptom grading registered before and during PS, a tool that would permit us to evaluate more objectively patient symptom burden and the efficacy of PS. In addition, albeit in our setting PS is most often administered continuously and using midazolam or neuroleptics as the main drugs, we did not study intermittent PS or other sedatives. This and the fact that we excluded ICU patients due to difficulties in distinguishing the real purpose of sedation for critical care patients in a retrospective study might have affected our PS prevalence. Finally, we acknowledge that comparing the time from admission to death between sedated and non-sedated patients is not a strong measure of survival. However, this is the only ethical and feasible way to do any comparison and to provide some information regarding this issue for providers, patients, and caregivers.

## Conclusion

PS is an ethical, recognized intervention that can be used to manage refractory symptoms at the end-of-life. It is a relatively common practice at our cancer center and it was not associated with shortening of hospital stay. Involvement of a dedicated palliative care team is strongly recommended if this procedure is being considered. Further research is needed to understand the efficacy of symptom relief with different sedatives and drug combinations and also to identify factors that may affect the frequency and outcomes associated with PS.

## Additional files

Additional file 1: Palliative Sedation Institutional Policy. All the institutional guidelines on PS is provided in this file. (PDF 410 kb)

Additional file 2: PS details. Details of palliative sedation are provided in this table. (DOCX 19 kb)

## Abbreviations

ICU: Intensive Care Unit
NAPCH: National Academy of Palliative Care Handbook
PS: Palliative sedation
SDU: Step Down Unit
