# Supplementary material for: Continuous palliative sedation for patients with advanced cancer at a tertiary care cancer center
Source: BMC Palliat Care. 2018 Jan 4;17:13. doi: 10.1186/s12904-017-0264-2 (PMC5755023; doi:10.1186/s12904-017-0264-2)
Supplement: Supplementary file 1 — Palliative Sedation Institutional Policy. All the institutional guidelines on PS is provided in this file. (PDF 410 kb) [file 12904_2017_264_MOESM1_ESM.pdf]

ALBERT EINSTEIN
SOCIEDADE BENEFICENTE ISRAELITA BRASILEIRA
HOSPITAL • ENSINO E PESQUISA • RESPONSABILIDADE SOCIAL

Tipo Documental
PoliticaTecnica
Título Documento
Sedação Paliativa

Sedação Paliativa

Definição

Define-se como sedação paliativa a redução intencional do nível de consciência, contínua ou intermitente com o objetivo de controlar sintomas causadores de grave desconforto refratários ao tratamento convencional, presentes na evolução de doenças incuráveis e progressivas em fase terminal.

A sedação paliativa não apresenta as mesmas definições e intenções da Eutanásia , suas diferenças caracterizam-se de acordo com a tabela abaixo (tabela-1):

	**Sedação Paliativa**	**Eutanásia**
**Meta**	**Aliviar o sofrimento**	**Aliviar o Sofrimento**
**Intenção**	**Sedar**	**Matar**
**Processo**	**Administração de droga sedativa, controle de sintomas**	**Administração de dose letal**

Diretoria<br>PRATICA MEDICA		Espécie<br>TECNICO	Especialidade<br>MEDICO	Status<br>Aprovado
Código Legado<br>GEPM.PO.CO.018	Código do Documento<br>PO.TEC.5.2	Versão<br>2	Data Criação<br>02/01/2009	Data Revisão<br>01/03/2012 00:00:00
Elaborador<br>Camila Sardenberg	Revisor<br>Camila Sardenberg	Parecerista	Aprovado por<br>Carla Souza Behr	Data Aprovação<br>01/03/2012 00:00:00

DOCUMENTO OFICIAL

**Evolução imediata**	**Diminuição do nível de conciência**	**morte**

Objetivos

1.Controlar sintomas de grave desconforto, refratários ao tratamento paliativo convencional, aliviando o sofrimento de pacientes em fase final de doença grave, progressiva e incurável.
2. Melhorar o conforto e manutenção da dignidade da vida humana até o seu final.

Indicação

É aplicável a todos os pacientes internados ou em assistência domiciliar do HIAE com diagnóstico de estado final de doença grave, progressiva e incurável, com sintomas refratários ao tratamento, que causem grave desconforto ao paciente. Os sintomas devem ser aliviados com prioridade são:

- Dispnéia
- Sangramento
- Dor
- Agitação e delirium terminal
- Náuseas e vômitos

- Fadiga severa com distúrbios de sono
- Outros sintomas que causem grande sofrimento ao paciente e que não sejam passíveis de controle paliativo convencional

Instruções Específicas

Critérios para indicação da sedação paliativa:

1.A doença é irreversível e a morte é esperada em poucas horas ou dias.

2.Os sintomas pelos quais se busca alívio são claramente definidos e compreendidos.

3.Os sintomas devem ser intoleráveis ao paciente. Situações de sofrimento familiar devem tratadas com suporte psicológico intensivo.

4.Os sintomas devem ser refratários ao tratamento e em caso de dúvidas, um profissional médico qualificado em Cuidados Paliativos deve ser consultado.

5.A decisão de sedação deve ser devidamente documentada em prontuário por toda equipe assistencial assim como também deve estar devidamente relatado em prontuário a evolução clínica subseqüente a sedação.

6.Esta decisão deve estar de acordo com a vontade do paciente, família ou responsável legal e em consenso com a equipe médica.

7.Contra indicações, metas e expectativas com relação á sedação devem ser discutidas com a família, equipe assistencial e com o paciente, quando possível.

8.Deve ser esclarecida a possibilidade de suspensão ou intermitência da sedação.

9.Membros da família devem ser encorajados a permanecer ao lado do paciente.

10. todas as pessoas envolvidas nos cuidados tem que estar ciente da condição clínica do paciente e das decisões tomadas pela equipe e família.

11.Os cuidados básicos higienização, controle de dor, manuseio de secreções, mobilização no leito devem ser mantidos e o protocolo de conforto deve ser imediatamente implantado.

12.Somente as medicações que promovam alívio de sofrimento devem ser mantidas.

13.Cuidados especiais com paciente e familiares devem ser tomados tais como um ambiente confortável, redução de ruídos desnecessários, sendo a privacidade da família e do paciente respeitada.

14.Suporte psicológico de apoio ao processo de luto deve ser oferecido a todos os pacientes e familiares com indicação de sedação paliativa. O suporte deve ser mantido após o óbito.

15.A equipe assistencial mantém a avaliação de riscos e necessidades considerando os aspectos espiritual, emocional, cultural e social respeitando as condições e solicitações da família. O suporte espiritual deve ser sempre oferecido.

16. Todas as etapas do processo de cuidado devem estar claramente descritas no prontuário do paciente.

17.Após o óbito do paciente, a equipe de cuidados envolvida deve ter a oportunidade de discutir seus sentimentos, dúvidas e experiências com relação ao paciente falecido.

Instruções Especiais

1. Em casos de discordância de conduta entre familiares, paciente e/ou equipe médica na indicação de sedação paliativa, o fluxo de gerenciamento de conflitos do HIAE.

Ao iniciar sedação sugere-se:
a. Revisar periodicamente o nível de sedação do paciente .

b. Descrever avaliação clínica no prontuário
c. Proporcionar sempre
Presença
Disponibilidade
Privacidade
Compreensão

Drogas sugeridas para sedação paliativa:
Os grupos farmacológicos de escolha estabelecidos pela literatura são (tabela 2):
Benzodiazepinicos: Midazolan é a droga de escolha com o benefício de poder ser utilizada por via sub cutânea e endovenosa
Neurolépticos: levomepromazina é a droga de escolha, tem ação mais sedativa que o haloperidol e pode ser usada via sub cutânea , considera-se o uso também da clorpromazina.
Barbituricos: droga de escolha fenobarbital, boa ação sedativa, para náusea e pode ser utilizado por via sub cutânea.
Anestésicos: propofol (requer equipe especializada e habilitada para sua administração).

Tabela-2

**Droga**	**Dose/via**	**Observações**

**Midazolan**	**diluir 50 ml de SF 0,9% /5-10 mg SC ou EV lento (esta dosagem pode ser repetida a cada 1 - 2 hs)<br>Contínuo 0.5-2.5mg/h SC ou EV**	**Se não apresentar efeito satisfatório com a dosagem de 20mg/h, optar pela associação de nova droga ou troca de sedativo**
**Clorpromazina ou Levomepromazine**	**Clorpromazine -25mg S C ou EV, caso necessário administrar 50 mg após 2 hs.<br>Levomepromazine 0.5/8 mg/h EV ou SC**	**Levomepromazina pode ser utilizado em associação ou isoladamente**
**Fenobarbital**	**Fenobarbital em bolus100-200 mg EV ou SC seguido de 40-60mg/h contínuo**	**Suspender os demais sedativos**
**Propofol**	**20-50 mg EV em bolus seguido de dose contínua 20 mg/h EV**	**Ação ultra rápida, Uso orientado por médico**

	**(dose máxima de10mg/h)**	**qualificado**

Observações:

1. Os opióides não devem ser utilizados isoladamente para obter sedação.
2. Meperidina é formalmente contra-indicada em uso isolado ou associado.
3. Os opióides prescritos devem ser mantidos com objetivo de alívio de dor e melhora dos sintomas respiratórios.
4. Deve ser mantido o uso de neurolépticos quando a sedação paliativa é introduzida devido ao quadro de *delirium / agitação* refratários.
5. Outras drogas sedativas podem ser utilizadas, desde que sob orientação de um profissional qualificado em Cuidados Paliativos

Registro

Prontuário do Paciente.
Rotina de registro e documentação de consenso com a família e paciente.

Anexos

Referências:

1. Fainsinger RL, deMoissac D, Mancini I, ones- chuk D. Sedation for et al. and other symp- toms in terminally ill patients in Edmonton. J Palliat Care. 2000;16:5-10.

2. Fainsinger RL, Waller A, Bercovici M et al.. A multicentre international study of sedation for uncontrolled symptoms in terminally ill patients. Palliat Med. 2000;14:257-265.
3. Cowan JD, Walsh D. Terminal sedation in palliative medicine - definition and review of the literature. Support Care Cancer. 2001; 9;403-407.
4. Chan KS, Shan MMK, Tse DMW, Thorsen AB. Palliative medicine in malignant respira- tory diseases. In Doyle D, Hanks G, Cherny N, Calman K. Oxford Textbook of Palliative Medicine. 3th Ed. Oxford University Press; 2004;(Cap. 8.8):587-618.
5. Morita T. Palliative sedation to relieve psycho- existential suffering of terminally ill can- cer patients. J Pain Symptom Manage. 2004;28:445-450.

Descrição Resumida da Revisão

Inserida tabela sobre as diferenças entre eutanásia e sedação paliativa.Revisadas doses e guidelines de drogas para sedação.

Diretoria<br>PRATICA MEDICA		Espécie<br>TECNICO	Especialidade<br>MEDICO	Status<br>Aprovado
Código Legado<br>GEPM.PO.CO.018	Código do Documento<br>PO.TEC.5.2	Versão<br>2	Data Criação<br>02/01/2009	Data Revisão<br>01/03/2012<br>00:00:00
Elaborador<br>Camila Sardenberg	Revisor<br>Camila Sardenberg	Parecerista	Aprovado por<br>Carla Souza Behr	Data Aprovação<br>01/03/2012<br>00:00:00
DOCUMENTO OFICIAL				
