# Supplementary material for: Continuous palliative sedation for patients with advanced cancer at a tertiary care cancer center
Source: BMC Palliat Care. 2018 Jan 4;17:13. doi: 10.1186/s12904-017-0264-2 (PMC5755023; doi:10.1186/s12904-017-0264-2)
Supplement: Supplementary file 2 — PS details. Details of palliative sedation are provided in this table. (DOCX 19 kb) [file 12904_2017_264_MOESM2_ESM.docx]

**Details of PS**

|  | All included patients | Midazolam only | Midazolam+neuroleptic | Neuroleptic only | p-value | Seen by palliative care | Not seen by palliative care | p-value |
| --- | --- | --- | --- | --- | --- | --- | --- | --- |
| **Total (%)** | 203 (100) | 107 (52.7) | 80 (39.4) | 16 (7.88) |  | 25 (12.3) | 179 |  |
| **Midazolam dose (median, IQR)** |  |  |  |  |  |  |  |  |
| **Initial** | 0.75 (0.6 - 1.5) | 0.8 (0.6-1.5) | 0.73 (0.6-1.34) | na | 0.3 | 0.6 (0.4-1.1) | 0.77 (0.6-1.5) | 0.11 |
| **Final** | 1.5 (0.9-3.0) | 1.7 (0.77-3.55) | 1.5 (1-2.9) | na | 0.5 | 1.2 (0.6-2.2) | 1.5 (1-3.4) | 0.09 |
| **Chlorpromazine dose (median, IQR)** |  |  |  |  |  |  |  |  |
| **Initial** | 1.4 (1-3) | na | 1.4 (1-2.5) | 2.5 (1-3.2) | 0.032 | 1 (0.5-2.05) | 1.41 (1-3) | 0.68 |
| **Final** | 2 (1-4) | na | 2 (1-4.2) | 2.5 (1-4) | 0.33 | 1.2 (1-2.05) | 2.0 (1-4.4) | 0.21 |
| **Opioid used** |  |  |  |  | 0.083 |  |  | 0.067 |
| **No opioids** | 4 (2%) | 2 (1.87%) | 1 (1.2%) | 5 (31.2%) |  | 0 (0%) | 0 (0%) |  |
| **Morphine** | 184 (93%) | 98 (91.63)% | 75 (93.9%) | 11 (62.9%) |  | 23 (92%) | 161 (94.15%) |  |
| **Fentanyl** | 9 (4.6%) | 7 (6.5%) | 2 (2.5%) | 0 (0%) |  | 1 (4%) | 8 (4.68%) |  |
| **Methadone** | 1 (0.5%) | 0 (0%) | 1 (1.2%) | 0 (0%) |  | 1 (4%) | 0 (0%) |  |
| **Other** | 2 (1%) | 0 (0%) | 1 (1.2%) | 1 (6.2%) |  | 0 (0%) | 2 (1.17%) |  |
| **Opioid dose used in the last 24hs (MEDD)** |  |  |  |  | 0.15 |  |  |  |
| **Mean (SD)** | 85 (96) | 94.2 (109.9) | 80.4 (82.0) | 41.8 (32.5) |  | 69.4 (71) | 87.6 (99.8) | 0.38 |
| **Median (IQR)** | 48 (24-105) | 49 (25.5-116.1) | 49 (24.5-105) | 38.4 (16.3-60) |  | 40 (24-64) | 48 (24-109) | 0.48 |
| **Other interventions** |  |  |  |  |  |  |  |  |
| **Steroids** | 64 (32%) | 32 (30.5%) | 27 (33.7%) | 5 (33.3%) | 0.88 |  |  | 0.067 |
| **IV fluids** | 62 (20.5%) | 33 (30.6%) | 22 (27.5%) | 7 (46.7%) | 0.33 |  |  | 0.119 |
| **Parenteral nutrition** | 28 (13.8%) | 18 (16.7%) | 7 (8.7%) | 3 (18.7%) | 0.05 |  |  | 0.85 |
| **Place of death - number (%)** |  |  |  |  | 0.25 |  |  |  |
| **Regular wards** | 168 (83%) | 85 (78.7%) | 70 (87.5%) | 14 (87.5%) |  |  |  |  |
| **Step-down unit** | 35 (17%) | 23 (21.3%) | 10 (12.5%) | 2 (12.5%) |  |  |  |  |
| **Time from admission to death (days)** | 33.6 (17-61.7) | 30.7 (17.2-51.3) | 37.8 (17-66.2) | 19.2 (8.2-64.8) | 0.29 |  |  |  |
| **Time from sedation to death (hours)** | 27 (5.5-66.2) | 27.9 (13.3-48.5) | 34.4 (13.1-66.2) | 8.66 (5.5-19.4) | 0.11 | 36.9 (17.6-52.5) | 26.1 (12.2-58) | 0.47 |
